# Occurrence and Genetic Diversity of *Trichomonas gallinae* in Captive Synanthropic Birds in Southeastern Brazil

**DOI:** 10.3390/pathogens15040428

**Published:** 2026-04-16

**Authors:** Amanda Garcia Pereira, Sarah Raquel Jesus Santos Simões, Maitê Cardoso Coelho da Silva, Ana Cláudia Calchi, Ricardo Bassini-Silva, Ana Carolina Castro-Santiago, Rosangela Zacarias Machado, Marcos Rogério André, Karin Werther

**Affiliations:** 1 Serviço de Patologia de Animais Silvestres—SEPAS, Departamento de Patologia, Reprodução e Saúde Única, Faculdade de Ciências Agrárias e Veterinárias, Universidade Estadual Paulista “Júlio de Mesquita Filho”—UNESP, Jaboticabal 14884-900, SP, Brazil; garcia.pereira@unesp.br (A.G.P.); sarahraqueljesusss@gmail.com (S.R.J.S.S.); maitecoelhocardoso@gmail.com (M.C.C.d.S.); 2 Vector-Borne Bioagents Laboratory—VBBL, Departamento de Patologia, Reprodução e Saúde Única, Faculdade de Ciências Agrárias e Veterinárias, Universidade Estadual Paulista “Júlio de Mesquita Filho”—UNESP, Jaboticabal 14884-900, SP, Brazil; ana.calchi@unesp.br (A.C.C.); rzacariasmachado@gmail.com (R.Z.M.); mr.andre@unesp.br (M.R.A.); 3 Laboratório de Coleções Zoológicas, Instituto Butantan, São Paulo 05503-900, SP, Brazil; ricardo.bassini@gmail.com; 4 Departamento de Medicina Veterinária Preventiva e Saúde Animal, Faculdade de Medicina Veterinária e Zootecnia, Universidade de São Paulo—USP, São Paulo 05508-270, SP, Brazil; ana_carolinacsantiago@hotmail.com

**Keywords:** wild birds, Columbiformes, culture, phylogeny, protozoa, *Trichomonas gallinae*

## Abstract

Avian trichomonosis is caused by protozoa of the genus *Trichomonas*, mainly *Trichomonas gallinae*, which infects the upper digestive tract of birds and is commonly associated with Columbiformes, the main reservoirs of the parasite. This study aimed to investigate the occurrence and genetic diversity of *Trichomonas* spp. in captive synanthropic birds from southeastern Brazil. Oropharyngeal swabs were collected from 281 birds belonging to 13 avian orders and analyzed using Diamond medium culture, Giemsa-stained smears, and molecular assays. Of the 262 samples submitted to culture analysis, 72 (27.48%) showed trophozoite-like structures under light microscopy. Molecular screening based on the ITS1–5.8S–ITS2 region detected Trichomonas DNA in 76 out of 267 samples with successful DNA extraction (28.46%), including 72 *Columba livia domestica* from Franca, one *Coragyps atratus* from Ribeirão Preto, and three rock doves from Jaboticabal. Among the ITS-positive samples, 67 (88.15%) amplified the *Fe*-*hydrogenase* gene, and 65 (85.5%) were also positive for the 18S rRNA gene. Only six samples (2.29%) exhibited structures compatible with *Trichomonas* spp. in Giemsa-stained smears. Phylogenetic analyses based on ITS sequences grouped the isolates into two clades within the *Trichomonas gallinae* complex. Greater genetic diversity was observed using *Fe-hydrogenase* and 18S rRNA markers, revealing multiple haplotypes and clades. Molecular assays, particularly PCR applied directly to oropharyngeal swabs, showed higher sensitivity for detecting and characterizing *Trichomonas gallinae* compared to culture and cytology. These findings highlight the high occurrence and genetic diversity of *T. gallinae* in captive synanthropic pigeons and reinforce the importance of molecular tools for epidemiological surveillance in wildlife facilities.

## 1. Introduction

Two main species of *Trichomonas* are known to infect birds: *Trichomonas gallinae* (Rivolta, 1878), which has major clinical significance among wild birds and typically colonizes the upper digestive tract (oropharynx, esophagus, and crop); and *Tetratrichomonas gallinarum* (Martin and Robertson, 1911), which primarily inhabits the lower digestive tract, especially the ceca of Galliformes and Anseriformes [1,2,3].

Although *T. gallinae* primarily infects the upper digestive tract, it may disseminate hematogenously to the lungs, liver, air sacs, pancreas, bones, and cranial sinuses [4]. Transmission occurs through several routes, including crop milk, courtship and mating behaviors, direct contact with infected individuals, and indirect exposure to contaminated food, water, or prey. Consequently, birds across a broad range of taxonomic orders may be infected, often asymptomatically [5,6]. In pigeons, which serve as principal reservoirs and disseminators of *T. gallinae*, early lesions manifest as small, white-to-yellowish plaques in the oral cavity. Severe infections may result in high mortality, particularly in young birds, with rates reaching 80–90% or higher [4].

Although direct diagnosis can be attempted using stained smears of oropharyngeal swabs, this method is often limited by the parasite’s fragility. Molecular techniques offer improved sensitivity and facilitate phylogenetic characterization of isolates. Polymerase chain reaction (PCR) is particularly effective at detecting low parasite loads. Several genetic targets, including the internal transcribed spacer (ITS) region, 18S rRNA, and *Fe-hydrogenase* genes, have proven valuable for species identification and haplotype discrimination [7]. Among these, ITS1 and ITS2 are widely used to confirm the presence of *T. gallinae* [8].

In Brazil, the earliest studies on avian *Trichomonas* infections were conducted in Rio Grande do Sul. De Carli et al. [9] and Tasca and De Carli [10] reported occurrence rates of 62.3% (104/167) and 26.47% (18/68), respectively, in domestic pigeons. Previous studies have used culture-based approaches for parasite detection and identification.

Arenales et al. [11] documented a case of trichomoniasis in a rock pigeon (*Columba livia domestica*) treated at the UNESP Veterinary Hospital in Araçatuba, São Paulo State. The bird died during clinical examination, and a white-yellowish, friable mass with a putrid odor was found on the hard palate. Cytological examination revealed protozoa consistent with *T. gallinae*. In another study, Joppert [12] reported characteristic oral lesions of trichomoniasis in raptors admitted to the Division of Veterinary Medicine and Wildlife Management in São Paulo. Lesions were observed in 10% (4/40) of caracaras (*Caracara plancus*), 10.8% (8/74) of American kestrels (*Falco sparverius*), and 4.2% (2/47) of Strigiformes (Tropical Screech-Owl—*Megascops choliba* and great horned owl—*Bubo virginianus*), with diagnosis based on saline wet-mount examinations. Additionally, Godoy et al. [13] detected compatible lesions in 1.66% (6/360) of Passeriformes submitted to the Wild Animal Rehabilitation Center at the Tietê Ecological Park.

In Minas Gerais State, Ecco et al. [14] examined paraffin-embedded tissues (tongue, oropharynx, esophagus, crop, proventriculus, and gizzard) and confirmed *Trichomonas* DNA in six birds by means of PCR targeting the intergenic and partial 5.8S rRNA regions. These included two Striped owls (*Asio clamator*), one American kestrel (*F. sparverius*), green-winged saltator (*Saltator similis*), and one toco toucan (*Ramphastos toco*). Phylogenetic analysis revealed two major clades. Sequences from a Striped owl and a kestrel grouped with *T. gallinae*, while one green-winged saltator sequence clustered with Simplomonas. The remaining two sequences formed separate clades. These findings suggest unresolved phylogenetic placements, later reassigned to *T. vaginalis-like* organisms based on further analysis of the 18S rRNA and α-tubulin genes [15]. In Rio Grande do Sul, Bruni et al. [16] confirmed *T. gallinae* in a chimango caracara (*Milvago chimango*) exhibiting characteristic lesions through culture and ITS1-based PCR.

Importantly, all birds included in this study were synanthropic species maintained in captivity in zoos, rehabilitation centers, or wildlife facilities and do not represent free-living urban populations.

Therefore, the present study aimed to (i) determine the occurrence of *Trichomonas* spp. in captive synanthropic birds from São Paulo State, Brazil, (ii) evaluate the genetic diversity of *T. gallinae* using ITS, *Fe-hydrogenase*, and 18S rRNA markers, and (iii) compare the performance of different diagnostic methods (culture, cytology, and PCR) for parasite detection.

## 2. Materials and Methods

### 2.1. Study Site and Samples

**Figure 1:**
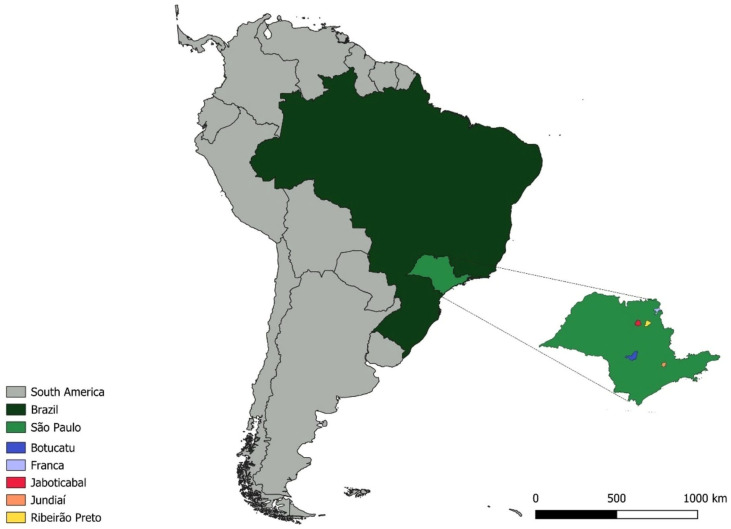
Collection sites highlighting Latin America, Brazil, and the state of São Paulo, with emphasis on the municipalities of Franca, Ribeirão Preto, Jaboticabal, Jundiaí, and Botucatu.

The oropharyngeal swab samples used (n = 281) in this study were collected between July 2022 and January 2023 from wild birds maintained in captivity in different cities (Table 1) in the state of São Paulo: Franca (Latitude: −20.5418, Longitude: −47.4197) (121 birds), Ribeirão Preto (Latitude: −21.170888, Longitude: −47.799273) (51 birds), Jundiaí (Latitude: −23.185705, Longitude: −46.897812) (58 birds), Botucatu (Latitude: −22.8904, Longitude: −48.4553) (32 birds), and Jaboticabal (Latitude: −21.2554, Longitude: −48.3224) (19 birds from the routine of SEPAS—Wild Animal Pathology Service) (Figure 1).

The birds included in this study originated from different captive conditions, including wildlife rehabilitation centers, zoos, and temporary holding facilities for rescued animals (Table 1). Most individuals were admitted due to injury or environmental displacement, and their duration of captivity varied from a few days to several months. These heterogeneous conditions may influence parasite exposure and transmission dynamics. From each of these birds, three oropharyngeal swabs were collected: (i) the first swab was stored in Diamond medium for the evaluation of trichomonad growth; (ii) the second was used to prepare Giemsa-stained smears on slides; and (iii) the third was used for DNA extraction and molecular assays.

For the remaining 19 birds that originated from the SEPAS routine, culture and Giemsa-stained cytology were not performed in Diamond medium because these birds were already dead and had been frozen.

### 2.2. Ethical Statement

All procedures were authorized by the Ethics Committees on Animal Use of the School of Agricultural and Veterinarian Sciences (FCAV/UNESP) (CEUA no. 732/21) and Institute Chico Mendes for Conservation of Biodiversity (SISBIO no. 77538-1).

### 2.3. Trichomonas spp. Culture

For the cultivation of *Trichomonas* spp., oropharyngeal swabs collected from captive birds were used.

Swabs intended for culture were immediately placed into Diamond medium, a modified version of the Trypticase–Yeast Extract–Maltose (TYM) medium. Each sample was resuspended in 9 mL of Diamond medium and incubated at 37 °C for 72 h. Cultures were examined daily for turbidity and for the presence of *Trichomonas* trophozoites using light microscopy. Prior to examination, samples were centrifuged at 8000 rpm for 15 min. A 10 µL aliquot of the pellet was placed on microscope slides and observed under a Eclipse E200 microscope (Nikon, Tokyo, Japan) at magnifications ranging from 40× to 1000×. Pellets from cultures showing growth were transferred to cryotubes and stored at −20 °C for subsequent DNA extraction and molecular analysis.

A total of 262 samples were subjected to culture in Diamond medium because some samples were obtained from birds that were already dead and frozen at the time of collection, which precluded culture-based analysis.

The term “trophozoite-like structures” refers to flagellated, motile organisms with morphology compatible with *Trichomonas* spp. under light microscopy.

### 2.4. Oropharyngeal Swab Smears

Oropharyngeal smears were prepared from birds sampled in zoos located in Ribeirão Preto (n = 51), Franca (n = 121), Jundiaí (n = 58), and Botucatu (n = 32). Smears were made on clean, degreased microscope slides by gently spreading the sample in a circular motion. After air drying, slides were fixed in absolute methanol for 3 min and stained with Giemsa solution (Interlab) for 40 min using approximately 5 mL per slide. Following staining, slides were rinsed with running water and allowed to dry vertically for 30 min before microscopic evaluation [17].

### 2.5. DNA Extraction

For DNA extraction, swabs were stored in 0.25 mL of autoclaved phosphate-buffered saline (PBS, pH 7.2) at −20 °C. Prior to extraction, samples were incubated at 40 °C for 15 min. No washing steps were performed before DNA extraction. Instead, excess PBS was removed, leaving ~300 µL of solution in the tube. Proteinase K and lysis buffer from the Biopur Mini Spin Plus kit (MoBius Life Science, Paraná, Brazil) were then added per the manufacturer’s instructions. The swab was removed before loading the lysate into the spin column, and the remainder of the protocol followed the kit guidelines.

Delays between sample collection and processing, as well as freezing of cultures, may have negatively affected DNA yield and PCR amplification efficiency.

### 2.6. Endogenous β-Actin PCR and DNA Electrophoresis in Agarose Gel

To assess the presence of PCR inhibitors and avoid false negatives, amplification of the endogenous avian β-actin gene was performed using conventional PCR with primers β-actin-F (5′-ATCTCGTCTTGTTTTATGCG-3′) and β-actin-R (5′-TATCCGTAAGGATCTGTATG-3′) [18]. Ultra-pure water was used as a negative control, and DNA from Pantanal birds [19] served as a positive control. PCR products were resolved via electrophoresis in a 1.0% agarose gel stained with ethidium bromide (0.5 µL/mL) in TBE buffer (pH 8.0), run at 100 V/150 mA for 50 min. A 100 bp molecular weight marker (Life Technologies^®^, Carlsbad, CA, USA) was used to estimate product size, visualized under a ChemiDoc MP Imaging System (Bio-Rad^®^, Hercules, CA, USA).

### 2.7. PCR Assay for Trichomonas spp. and Subsequent Sequencing and BLASTn Analysis

Samples that tested positive for the β-actin gene and those from positive cultures were screened for *Trichomonas* DNA using conventional PCR targeting the ITS1/5.8S/ITS2 region (369 bp) [20]. Positive samples were further characterized via PCR amplification of the near-full-length 18S rRNA gene (~1500 bp) [21] and the *Fe-hydrogenase* gene (~1000 bp) [22]. Table 1 lists the primers and cycling conditions. Ultra-pure water and DNA from *T. gallinae* isolated from a naturally infected *Falco peregrinus* were used as negative and positive controls, respectively. The primer sequences and thermal cycling conditions used for each PCR assay (ITS1/5.8S/ITS2, 18S rRNA and *Fe-hydrogenase* genes) are described in Table 2.

To obtain extended sequences of the 18S rRNA gene, additional primers were designed (F: 5′-CAAGGGCGAGAGTAGGAGTA-3′, R: 5′-ACCGAGTCATCCAATCGGTA-3′), which were used alongside standard primers for sequencing.

Consensus sequences were constructed using Phred-Phrap v23 [23] from bidirectional reads, with a minimum base quality score of 20. Sequence identity was assessed by BLASTn searches against GenBank (https://www.ncbi.nlm.nih.gov/genbank (accessed on 12 January 2026)).

### 2.8. Phylogenetic Analyses

Sequences were aligned with reference sequences retrieved from GenBank using ClustalW in BioEdit v7.0.5.3 [24]. Bayesian phylogenetic trees were generated using MrBayes v3.2.2 on XSEDE [25] via the CIPRES Science Gateway [26]. Analyses were run for 1,000,000 generations, with evolutionary models selected via jModelTest 2 [27]. Tree editing and rooting using outgroup sequences were conducted in TreeGraph v2.0.56-381 beta [28].

### 2.9. Diversity Analysis and Haplotype Network

New alignments were constructed using the ITS1/5.8S/ITS2, the near-full-length 18S rRNA and the *Fe-hydrogenase* nucleotide sequences of *T. gallinae* obtained in the present study, along with other sequences retrieved from GenBank and derived from birds sampled in different countries. For this purpose, only sequences longer than 321 bp for the ITS region, 1351 bp for the 18S rRNA gene, and 851 bp for the *Fe-hydrogenase* gene were selected. These three alignments were used in the genetic diversity analysis, conducted with DnaSP v5 software [29], to calculate nucleotide diversity (π), haplotype diversity (Hd), the number of haplotypes (h), and the average number of nucleotide differences (K). TCS networks were generated with popART (https://popart.maths.otago.ac.nz/ (accessed on 12 January 2026)) [30] to visualize haplotype relationships based on the different genetic markers.

## 3. Results

### 3.1. Trichomonas spp. Culture

A total of 262 samples were included in the culture analysis. Although 281 oropharyngeal swabs were collected in total, 19 samples originated from birds obtained through the SEPAS routine and had been previously frozen at the time of collection. As freezing compromises the viability of *Trichomonas* trophozoites, these samples were not suitable for culture-based analysis and were therefore excluded.

**Figure 2:**
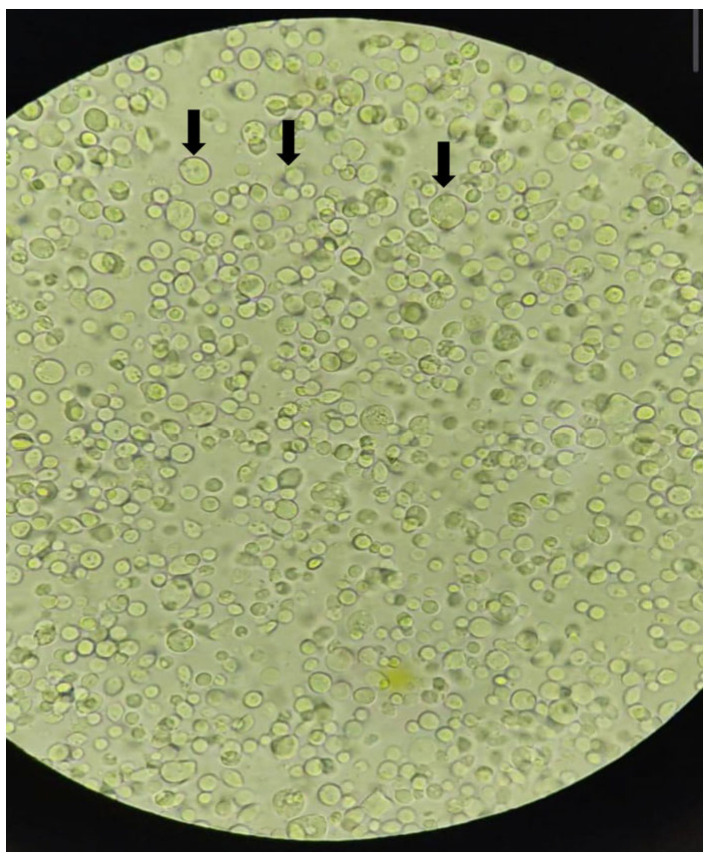
Microscopic examination of fresh culture of *Trichomonas* sp., (40×), at the arrow showing pseudocysts of *Trichomonas* sp.

Of the 262 samples submitted to Diamond medium culture, 72 (27.48%) showed structures consistent with *Trichomonas* trophozoites under light microscopy, exhibiting characteristic motility (Figure 2) (see Supplementary Videos S1 and S2). DNA extraction was performed using the Tissue kit (Qiagen, Valencia, CA, USA) for subsequent *Trichomonas* spp.-specific PCR testing, which revealed that only cultured samples from *C. livia* were positive. Additionally, two samples from Columbiformes from the same location were positive for the *Fe*-*hydrogenase* gene, while no sample amplified the 18S rRNA gene.

### 3.2. Giemsa-Stained Oropharyngeal Swab Smears

**Figure 3:**
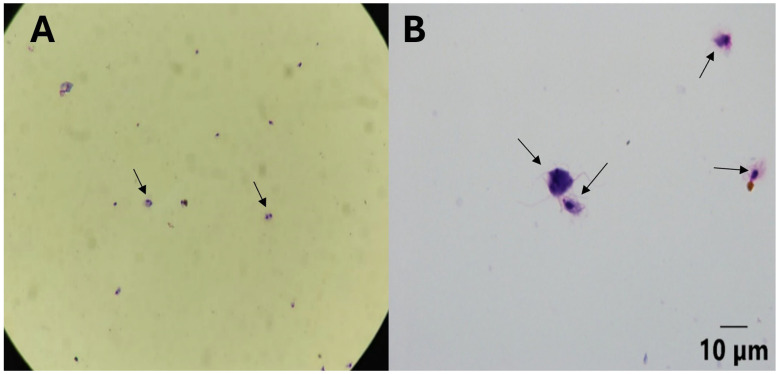
Trophozoites of *Trichomonas* sp. (black arrow) in Giemsa stained preparations (40× (**A**) and 100× (**B**)).

Among 262 Giemsa-stained oropharyngeal swab smears analyzed, six samples (2.29%) from Columbiformes in Franca revealed pseudocysts of *Trichomonas* spp. (Figure 3). These samples were negative in culture. Most smears showed varying quantities of anucleated and nucleated squamous cells, while seven samples (2.67%) also exhibited moderate numbers of red blood cells.

None of the samples positive by Giemsa-stained smears were exclusively detected by this method; all represented a minority subset compared to molecular and culture-based detection.

### 3.3. DNA Extraction and PCR for Endogenous Gene

DNA was extracted from 281 oropharyngeal swabs collected from captive birds. Of these, 267 (95.01%) tested positive for the endogenous avian *β-actin* gene.

### 3.4. PCR and Sequence Analysis of the ITS1/5.8S/ITS2 Regions of Trichomonas spp.

Out of the 267 samples positive for the endogenous avian *β-actin* gene, 76 (28.46%) tested positive in the PCR assay based on the ITS1/5.8S/ITS2 regions of *Trichomonas*, comprising: 72/121 rock doves from Franca, 1/5 *C. atratus* from Ribeirão Preto, and 3/12 rock doves from Jaboticabal. Detailed individual results for all samples are provided in Supplementary Table S1.

From the 76 positive samples, 34 amplicons with high intensity bands in agarose gel electrophoresis were sequenced. BLASTn analysis showed that the obtained ITS1/5.8S/ITS2 sequences had identities ranging from 93.73% to 100% with sequences of *T. gallinae* detected in *C. livia* in Spain (EU881912) and *C. livia* in China (MH733819) (Supplementary Table S2).

**Figure 4:**
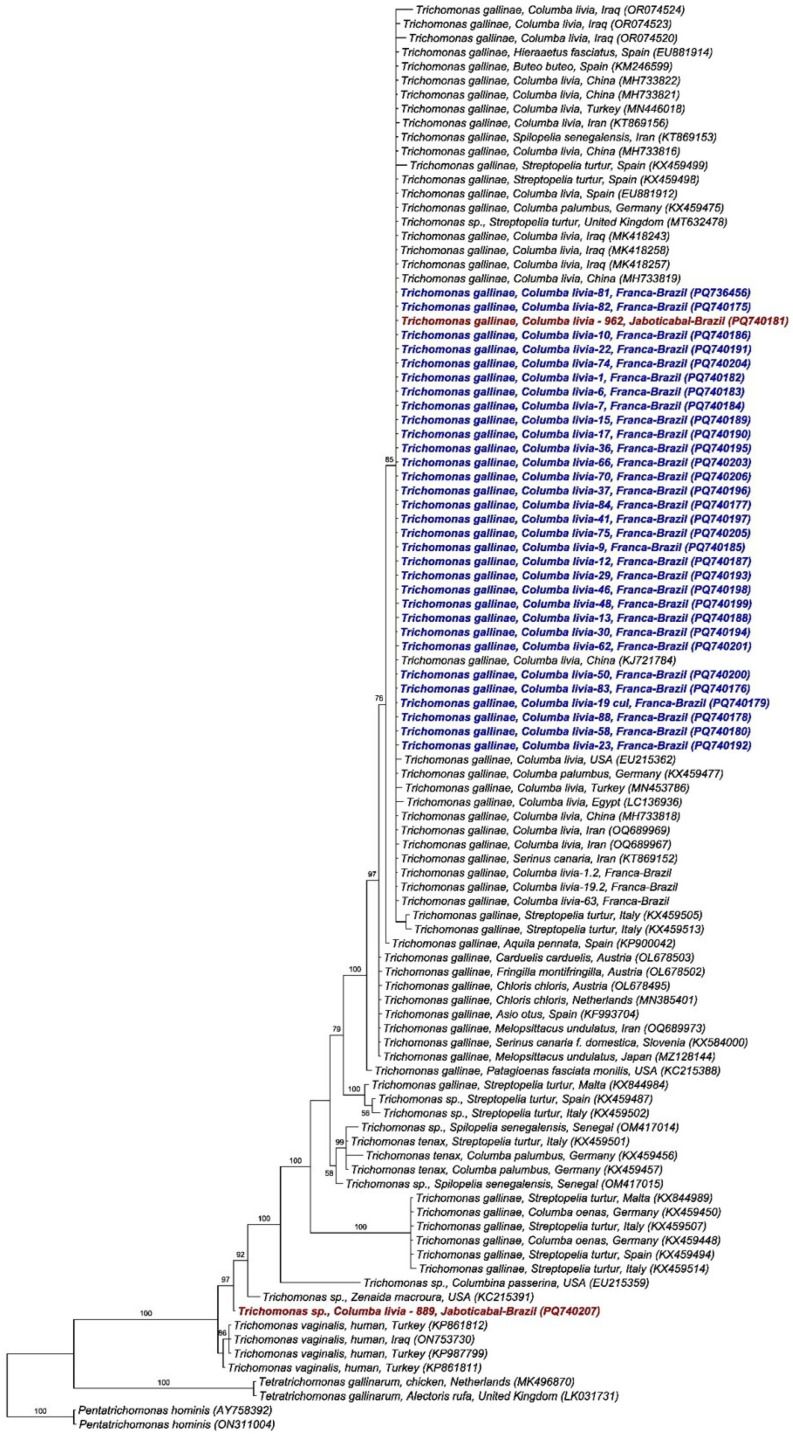
Phylogenetic analysis inferred by the Bayesian method and TIM2 + G evolutionary model of a 399 bp alignment of ITS1/5.8S/ITS2 sequences of *Trichomonas* spp. The sequences obtained in the present study are highlighted in color, with red corresponding to sequences obtained from birds sampled in Jaboticabal-SP, and blue to those obtained in Franca-SP. *Pentatrichomonas hominis* was used as an outgroup.

Phylogenetic analysis using the Bayesian method and the TIM2 + G evolutionary model, based on a 399 bp alignment of the ITS1/5.8S/ITS2 regions (Figure 4) of *Trichomonas* spp., positioned the 34 *T. gallinae* sequences obtained in this study into two distinct clades. All sequences obtained from rock doves in Franca and one from Jaboticabal clustered in a single clade with posterior probability of 85%, along with *T. gallinae* sequences detected in Columbiformes sampled in China, Turkey, Iraq, Egypt, USA, Spain, Italy, Germany, and Iran, as well as Accipitridae (*H. fasciatus* and *B. buteo*) sampled in Spain and a Passeriforme (*S. canaria*) from Iran. Meanwhile, one sequence obtained from a pigeon in Jaboticabal was positioned in a distinct clade, closely related to *Trichomonas* sp. Detected in *Zenaida macroura* from the USA.

**Figure 5:**
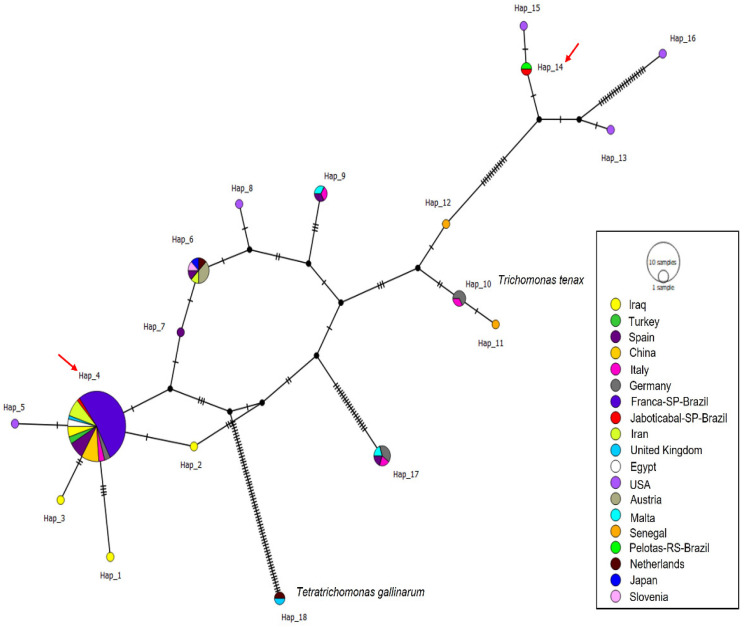
Haplotype network based on the ITS1-5.8S-ITS2 regions of *Trichomonas* spp. The colors represent the locations from which the sequences used in the analyses were detected, the black dots represent median vectors (inferred ancestral nodes), and the vertical black lines represent the number of mutational events between each haplotype. Red arrows indicate the haplotypes (#4 and #14) that contain sequences obtained in this study.

A total of 96 ITS1/5.8S/ITS2 sequences were analyzed (34 sequences obtained in this study and 62 *Trichomonas gallinae* sequences retrieved from GenBank), resulting in 18 distinct haplotypes. The sequences obtained in this study were distributed into only two haplotypes (haplotypes #4 and #14). Haplotype #4 was separated from haplotypes #2 and #5 by just one mutational event and comprised all sequences detected in Franca, along with one sequence from Jaboticabal and others detected in *C. livia*, *S. turtur*, *C. palumbus*, and *H. fasciatus* sampled in Iran, China, the UK, Spain, Turkey, and Egypt. Haplotype #14 was separated from haplotype #15 by just one mutational event and included one sequence from Jaboticabal and one detected in *Ramphastos dicolorus* from Pelotas, RS (Figure 5).

The diversity data are presented in Table 3. Haplotype numbering is locus-specific and does not imply correspondence among ITS1/5.8S/ITS2, *Fe-hydrogenase*, and 18S rRNA markers.

### 3.5. PCR and Analysis of the Fe-Hydrogenase Gene Sequences of Trichomonas spp.

For the *Fe-hydrogenase* gene, of the 76 samples analyzed, 67 (88.15%) were positive and sequenced, all from rock doves sampled in Franca. BLASTn analysis showed that the obtained sequences presented identities ranging from 98% to 100% with sequences of *T. gallinae* detected in *Streptopelia decaocto* in Spain (Supplementary Table S2).

**Figure 6:**
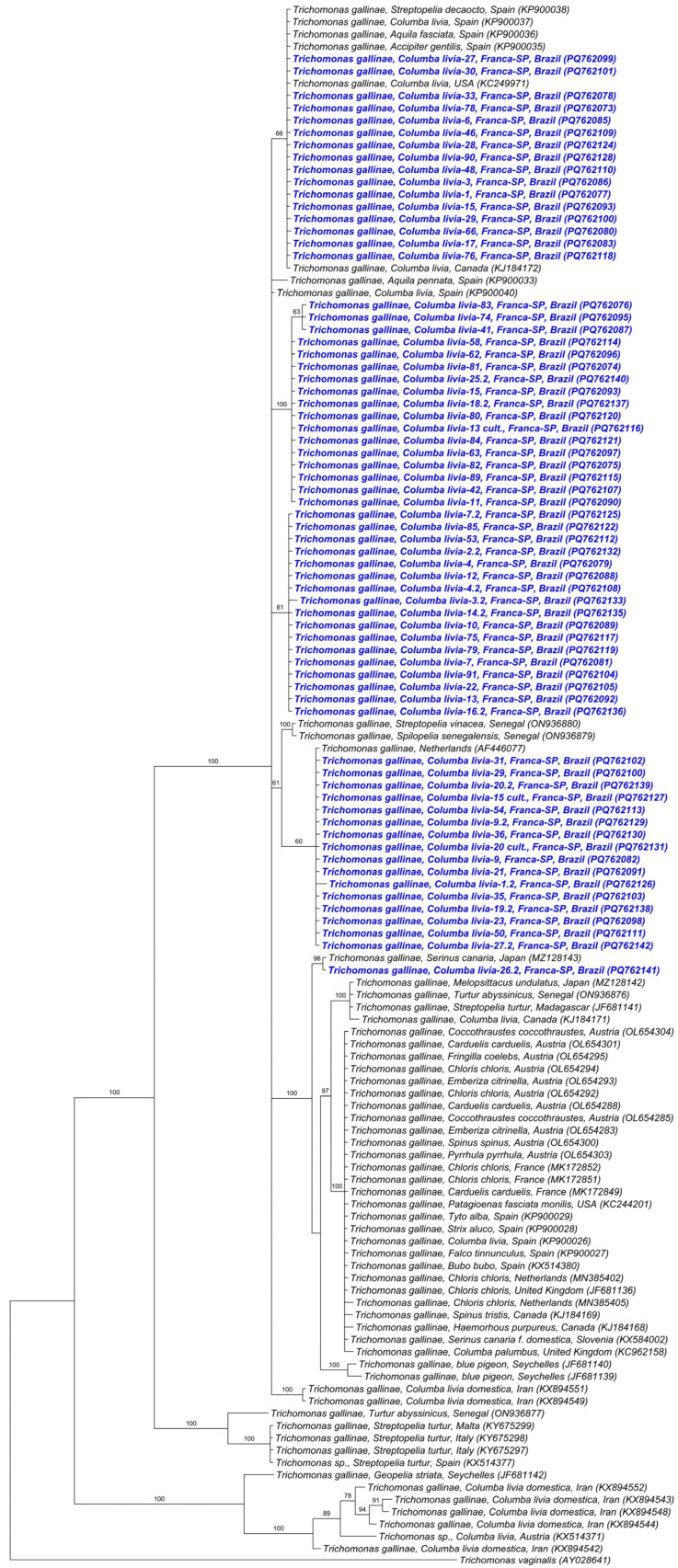
Phylogenetic analysis inferred by the Bayesian method and TIM2 + I + G evolutionary model of an alignment of 879 bp of *Fe-hydrogenase* gene sequences from *Trichomonas* spp. The sequences obtained in the present study are highlighted in blue. *Trichomonas vaginalis* was used as an outgroup.

Phylogenetic analysis using the Bayesian method and the TIM2 + I + G evolutionary model from an 879 bp alignment of the *Fe*-*hydrogenase* gene (Figure 6) showed that the *Fe-hydrogenase* sequences obtained in the study were distributed among several clades, with support indices varying from 60% to 100%. Two clades were composed solely of sequences detected in Franca-SP. In one clade, the sequences clustered with sequences detected in Columbiformes (*C. livia*—USA, Canada, *S. decaocto*—Spain) and Accipitridae (*A. fasciata*—Spain, *A*. *gentilis*—Spain). Another clade was formed by sequences from the present study and one sequence of *T. gallinae* detected in the Netherlands. The last clade consisted of a sequence detected in Franca and another detected in a Passeriforme (*S. canaria*) in Japan.

**Figure 7:**
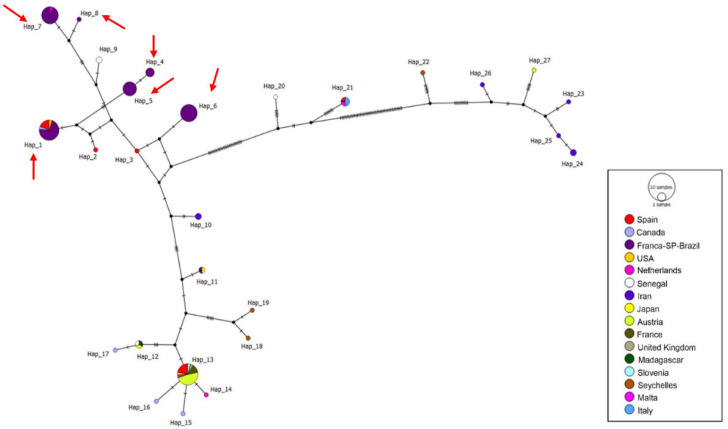
Haplotype network based on the *Fe-hydrogenase* gene of *Trichomonas* spp. The colors represent the locations from which the sequences used in the analyses were detected, the black dots represent median vectors (inferred ancestral nodes), and the vertical black lines represent the number of mutational events between one haplotype and another. Red arrows indicate the haplotypes (#1, #4 to #8) that contain sequences obtained in this study.

A total of 118 *Fe-hydrogenase* sequences were included in the haplotype analysis, comprising sequences obtained in this study (n = 60) and additional reference sequences retrieved from GenBank (n = 58). A total of 27 different haplotypes were obtained, and the sequences detected in this study were distributed among six distinct haplotypes: #1, #4, #5, #6, #7, and #8. Three haplotypes (#4, #5, and #6) were composed solely of samples from Franca, SP. Haplotype #1 was formed by 15 sequences from Franca, along with sequences detected in *S. decaocto*, *C. livia*, *A.* gentilis, and *R. pigeon* in Spain, Canada, and the USA, respectively. Haplotype #7 was formed by 14 sequences obtained from rock doves in Franca and sequences detected in *C. chloris* from Amsterdam (Figure 7). All haplotypes containing sequences from the present study, except for haplotype #4, which separated from haplotype #5 via a mutational event, were separated from median vectors by a mutational event. Haplotype numbering is locus-specific and does not imply correspondence among ITS1/5.8S/ITS2, *Fe-hydrogenase*, and 18S rRNA markers.

### 3.6. PCR and Sequence Analysis of the 18S rRNA Gene of Trichomonas spp.

Of the 76 analyzed samples, 65 (85.5%) were positive for the 18S rRNA gene, all from *C. livia* sampled in Franca. Of these, only seven samples were suitable for sequencing.

BLASTn analysis showed that the obtained sequences presented identities ranging from 99% to 100% with sequences of *T. gallinae* detected in *E. dove* from the USA and *C. livia* from Portugal (Supplementary Table S2).

**Figure 8:**
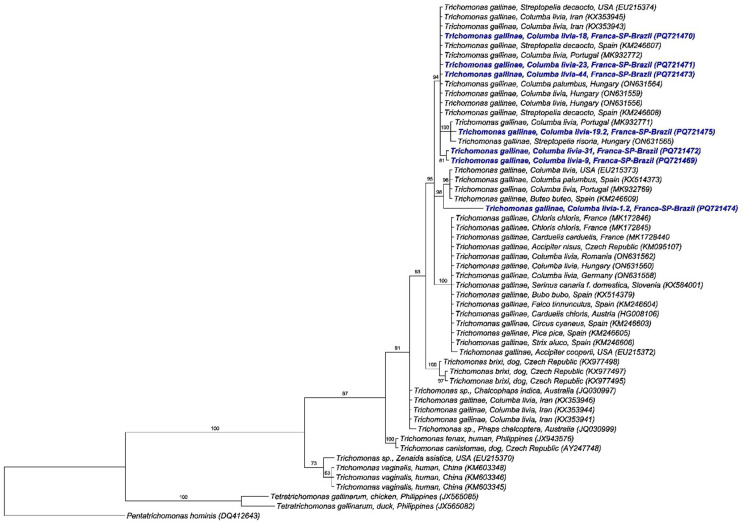
Phylogenetic analysis inferred by the Bayesian method and GTR + I + G evolutionary model of a 1424 bp alignment of 18S rRNA gene sequences of *Trichomonas* spp. The sequences obtained in the present study are highlighted in blue. *Pentatrichomonas hominis* was used as an outgroup.

Phylogenetic analysis using the Bayesian method and the GTR + I + G evolutionary model based on a 1424 bp alignment of the 18S rRNA gene (Figure 8) positioned the sequences obtained in this study into four distinct clades, with posterior probability values ranging from 91% to 100%. Two clades were composed solely of sequences detected in rock doves from Franca, with one formed by a single sequence that clustered near a clade containing sequences of *T. gallinae* detected in *C. livia*, *C. palumbus*, and *B. buteo* in the USA, Spain, and Portugal. The other two clades were composed of sequences from this study alongside sequences detected in Columbiformes from Iran, Hungary, Portugal, Spain, and the USA.

**Figure 9:**
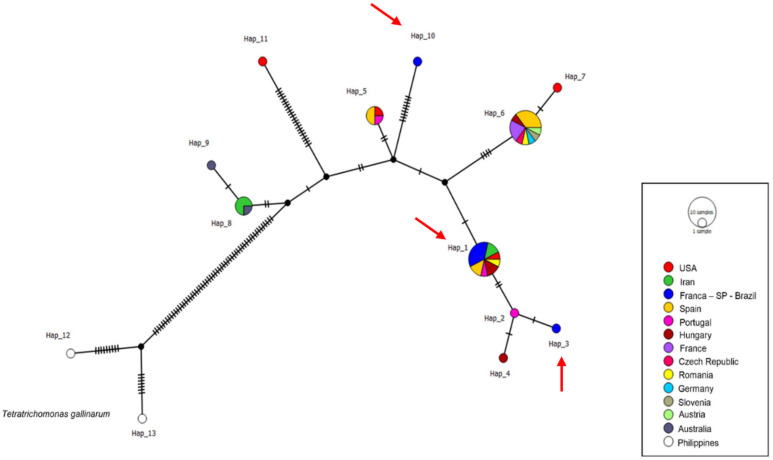
Haplotype network based on the 18S rRNA gene of *Trichomonas* spp. The colors represent the locations from which the sequences used in the analyses were detected, the black dots represent median vectors (inferred ancestral nodes), and the vertical black lines represent the number of mutational events between one haplotype and another. Red arrows indicate the haplotypes (#1, #3 and #10) that contain sequences obtained in this study.

In the haplotype analysis, 45 sequences of the 18S rRNA gene were used (seven obtained in the present study and 38 retrieved from GenBank), which were distributed across 13 different haplotypes. The sequences detected in this study comprised three distinct haplotypes (#1, #3, and #10). Haplotype #1 was composed of 5 sequences from Franca, along with sequences detected in *C. livia, S. decaocto*, and *C. palumbus* in Spain, Portugal, Hungary, Romania, the USA, and Iran. Haplotypes #3 and #10 were composed solely of sequences obtained from *C. livia* sampled in Franca. Haplotype #10 separated from a median vector by nine mutational events (Figure 9). Haplotype numbering is locus-specific and does not imply correspondence among ITS1/5.8S/ITS2, *Fe-hydrogenase*, and 18S rRNA markers.

## 4. Discussion

The primary hosts of *T. gallinae* are Columbiformes, particularly rock doves (*C. livia*), which likely explain the high number of positive swab samples obtained from the upper digestive tract in this study [31]. Avian trichomoniasis is a parasitic disease affecting a wide range of bird species, including both wild and domestic birds globally. Most of the positive birds in our study shared similar feeding habits, such as frugivory, granivory, or insectivory [32]. Shared feeding behaviors may facilitate food contamination and subsequent transmission of the parasite, especially when infected birds feed near others [33]. This scenario was observed in the present study, as rock doves from Franca, which tested positive, were housed together in a shared enclosure. Notably, these were carrier pigeons with scheduled departures and returns, during which they had direct contact with each other and shared food and water sources. Their transportation for racing events may also promote the spread of the parasite by exposing other birds to contaminated environments.

No clinical signs or visible lesions were observed in the birds sampled in this study. However, trichomoniasis-associated lesions in the upper respiratory tract have previously been documented in passerines such as *Haemorhous mexicanus* in Bakersfield, California [34,35]. Clinical signs described in infected birds include weight loss, ruffled feathers, vomiting, dyspnea, dysphagia, and diarrhea [36]. Asymptomatic infections are possible and may be associated with avirulent strains or host factors such as immune competence, particularly in adult birds [37].

Previous studies have reported variable prevalence of *Trichomonas gallinae* in Columbiformes, depending on host species, geographic region, and diagnostic approach. For example, prevalence values of 34.2% in *Columba palumbus* in Spain [38] and 52.7% in *Columba livia domestica* in Spain [39] have been described. Lower prevalence rates were reported in mourning doves (*Zenaida macroura*) in Florida (5.6%) [40] and in white-winged dove (*Zenaida asiatica*) in the USA (21.3%) [41], whereas localized outbreaks with higher occurrence, such as 33.3% in *C. palumbus* in southern Spain, have also been documented [42].

In the present study, considering molecular positivity by at least one PCR target (ITS1/5.8S/ITS2, *Fe-hydrogenase* or 18S rRNA), *Trichomonas gallinae* showed a high occurrence in *C. livia domestica* from Franca. This finding likely reflects intense parasite circulation within a captive population, where host density, shared enclosures, and management conditions differ substantially from those of free-living birds, limiting direct quantitative comparisons with field-based prevalence studies.

Most sequences clustered with haplotypes previously reported in Columbiformes, Falconiformes, and Passeriformes, reinforcing the role of Columbiformes as the main reservoirs and sources of infection for other avian orders. The identification of a distinct clade composed of a single sequence from Jaboticabal suggests the occurrence of local genetic variation and potential host-associated structuring.

Broader phylogenetic analyses based on the *Fe-hydrogenase* and 18S rRNA genes revealed a higher level of genetic diversification compared to ITS-based analysis, with six and four clades identified, respectively. This greater resolution highlights the importance of using multiple molecular markers for a more comprehensive assessment of genetic diversity within the *T. gallinae* complex. In particular, the higher haplotype variability observed for the *Fe-hydrogenase* gene may reflect its greater discriminatory power compared to ribosomal or intergenic regions [43].

The presence of multiple haplotypes circulating within the same host population suggests the coexistence of genetically distinct lineages, which may have implications for pathogenicity, transmission dynamics, and host susceptibility.

The presence of diverse haplotypes and clade distributions is well supported in the literature. Gerhold et al. [15] identified 12 different ITS1-5.8S-ITS2 sequence groups in *Trichomonas* spp., and Grabensteiner et al. [44] highlighted genetic heterogeneity within *T. gallinae* using *Fe-hydrogenase* and ITS markers. Further research is necessary to determine whether these genetic variants represent a single species with multiple lineages or distinct species.

Culture has traditionally been considered a reference method for detecting *Trichomonas* spp. due to its interpretability and historical use, even in asymptomatic hosts [45]. Culture-based detection is considered more sensitive than direct smear microscopy using saline [22,46,47].

According to Diamond [48], the inclusion of fetal bovine serum is essential for parasite growth due to its content of amino acids, fatty acids, and trace elements. In this study, culture was more sensitive than Giemsa-stained smears, consistent with previous reports, [48,49]. However, PCR-based methods demonstrated higher sensitivity than both culture and cytology when applied directly to oropharyngeal swabs. Real-time PCR assays may further improve sensitivity and allow quantification of parasite load, representing a promising approach for future epidemiological studies [50,51].

Incubation time is critical for culture efficacy [45]. In our study, parasite growth was not observed at 24 h but became evident after 48 to 60 h, with optimal detection at 72 h. Without nutrient replenishment, protozoa perished beyond this point. Early attempts at DNA extraction after 72 h culture incubation faced challenges due to freezing, which negatively impacted yield. Immediate DNA extraction after culture led to improved detection, though PCR positivity remained lower than the number of culture-positive samples (72/262; 27.48%). This discrepancy may be attributed to PCR inhibition caused by components in the culture medium.

Only 6 of 262 oropharyngeal smears (2.29%) revealed *T. gallinae* trophozoites, confirming the low sensitivity of this method compared to culture and molecular diagnostics. The trophozoites observed were morphologically consistent with those described by Mohamed et al. [45].

The higher sensitivity observed for PCR compared to culture and cytology is consistent with previous studies, reinforcing its value as the preferred diagnostic method for detecting *Trichomonas gallinae*, especially in samples with low parasite load. Additionally, the use of multiple molecular markers allowed a more comprehensive assessment of genetic diversity, revealing the presence of multiple haplotypes circulating within the same host population.

The absence of a washing step prior to DNA extraction may have influenced PCR performance, possibly due to the presence of inhibitors derived from the culture medium or biological material. This factor may partially explain discrepancies between culture positivity and PCR amplification observed in this study. In addition, real-time PCR (qPCR) has been increasingly explored in avian pathogen detection due to its higher sensitivity and ability to quantify nucleic acids. This quantitative capacity may provide important insights into infection dynamics, including the relationship between parasite burden and clinical manifestation, as well as transmission potential in captive populations. Although not employed in the present study, the incorporation of real-time PCR in future investigations could improve diagnostic accuracy and contribute to a better understanding of the epidemiology of *T. gallinae.*

Besides *T. gallinae*, other species such as *T. vaginalis-like* organisms have been reported in doves and owls [14,15], while *T. tenax* has been identified in European pigeons [52]. *T. gypaetinii* was found in vultures and eagles [53,54]. Emerging variants such as *T. stlaberi* and *T. canistomae*-like have also been detected in *S. turtur* and *A. gentilis* in Spain, suggesting even greater diversity among avian trichomonads.

Given the confirmed role of Columbiformes as primary hosts, pigeons serve as the main reservoir for *T. gallinae* in many regions of São Paulo State [11,12]. Zoos, rehabilitation centers, and wildlife facilities should evaluate the risks associated with releasing birds that have recovered from infection, as asymptomatic carriers can serve as long-term reservoirs, facilitating the dissemination of the parasite.

## 5. Conclusions

A high occurrence of *T. gallinae* was found in pigeons in the present study, confirming the role of these animals as primary hosts for the agent. PCR assays showed higher sensitivity in this study, particularly when applied directly to oropharyngeal swabs, proving useful for both detection and molecular characterization of *Trichomonas gallinae*. The analysis of oropharyngeal swab smears from birds, while quick and easy to perform, showed low sensitivity for diagnosing *T. gallinae* infection and should not be used as the sole laboratory method for diagnosing trichomoniasis. Although the culture of the parasite in Diamond medium shows good sensitivity for detection, the sample must be kept in this medium and incubated at 37 °C immediately after collection, which hampers the use of this technique in epidemiological studies in remote locations. Different haplotypes of *T. gallinae* occur in rock doves in the southeastern region of Brazil. The *Fe-hydrogenase* gene proved to be the molecular marker with the greatest capacity to differentiate *T. gallinae* haplotypes when compared to the ITS-1-5.8S-ITS2 and 18S rRNA regions.

## Figures and Tables

**Table 1:** Description of sampled birds from different orders that were sampled in Jaboticabal, Franca, Ribeirão Preto, Botucatu and Jundiaí (SP, Brazil) for detection of *Trichomonas* spp. in oropharyngeal swabs.

City	Quantity	Order	Quantity/Species
Jaboticabal	12	Columbiformes	12 *Columba livia domestica*
	3	Piciformes	3 *R. toco*
	1	Strigiformes	1 *Tyto furcata*
	2	Psittaciformes	1 *A. arauna,* 1 *F. xanthopterygius*
	1	Charadriiformes	1 *Vanellus chilensis*
Franca	121	Columbiformes	121 *Columba livia domestica*
Ribeirão Preto	5	Accipitriformes	4 *R. magnirostris* e 1 *F. sparverius*
	5	Cathartiformes	5 *C. atratus*
	10	Columbiformes	10 *Columbina talpacoti*
	1	Nyctibiiformes	1 *Nyctibius griseus*
	10	Passeriformes	3 *Saltator similis*, 1 *Cacicus cela*, 3 *Turdus rufiventris*, 1 *Thraupis sayaca*, 1 *Mimus saturninus*, 1 *Pitangus sulphuratus*
	2	Piciformes	2 *R. toco*
	8	Psittaciformes	1 *A. arauna*, 3 *A. auricapillus*, 1 *Eupsittula aurea*, 3 *Pyrrhura frontalis*
	10	Strigiformes	5 *T. furcata*, 4 *A. cunicularia*, 1 *Megascops choliba*
Botucatu	19	Passeriformes	1 *Sicalis flaveola*, 2 *Oryzoborus maximiliani*, 2 *Cyanocompsa brissonii*, 2 *C. cucullatus*, 1 *O. angolensis*, 1 *S. similis*, 1 *Gnorimopsar chopi*, 3 *Sporophila caerulescens*, 2 *P. sulphuratus*, 2 *Tachornis squamata*, 1 *Thraupis sayaca* and 1 *Guira guira*
	2	Columbiformes	2 *Zenaida auriculata*
	3	Piciformes	3 *R. toco*
	1	Psittaciformes	1 *Brotogeris chiriri*
	1	Anseriformes	1 *Anas bahamensis*
	6	Strigiformes	4 *Ciccaba virgata*, 1 *Tyto furcata* 1 *Athene cunicularia*
Jundiaí	11	Piciformes	11 *R. toco*
	1	Nyctibiiformes	1 *Nyctibius griseus*
	10	Strigiformes	6 *C. virgata*, 2 *A. cunicularia*, 1 *Asio clamator*, 1 *P. perspicillata*
	11	Columbiformes	2 *Zenaida auriculata*, 3 *C. livia*, 6 *Columbina talpacoti*
	12	Falconiformes	2 *C. plancus*, 7 *Falco sparverius*, 1 *Ictinia plumbea*, 2 *Rupornis magnirostris*
	4	Cathartiformes	4 *C. atratus*
	2	Pelecaniformes	1 *Tigrisoma lineatum*, 1 *Ardea cocoi*
	1	Tinamiformes	1 *Rhynchotus rufescens*
	6	Psittaciformes	4 *Psittacara leucophthalmus*, 1 *Brotogeris tirica*, 1 *Amazona aestiva*

**Table 2:** Description of the primers and thermal conditions used in the conventional PCR assays for the endogenous gene and *Trichomonas* spp.

Primer Names	Primer Sequences	Thermal Conditions	References	Fragment Size
Endogenous gene				
β-actin-F	5′-CCTCATGAAGATCCTGACAGA3′	95 °C for 5 min, followed by 35 cycles at 95 °C for 30 s, 54 °C for 30 s and 72 °C for 1 min and final extension at 72 °C for 5 min	[18]	700 bp
β-actin-R	5′-TCTCCTGCTCYAAYTCCA-3′			
ITS1/5.8S/ITS2				
TRF1	(5′-TGCTTCAGCTCAGCGGGTCTTCC-3′)	94 °C for 2 min followed by 40 cycles at 94 °C for 20 s, 66 °C for 20 s and 72 °C for 30 s, final extension at 72 °C for 5 min	[20]	369 bp
TRF2	(5′-CGGTAGGTGAACCTGCCGTTGG-3′)			
18S rRNA				
Hm-Long-f	(5′-AGGAAGCACACTATGGTCATAG-3′)	95 °C for 15 min, followed by 40 cycles of 94 °C for 30 s, 55 °C for 1 min and 72 °C for 2 min, final extension at 72 °C for 10 min	[21]	1500 bp
Hm-Long-r	(5′-CGTTACCTTGTTACGACTTCTCCTT-3′)			
*Fe-hydrogenase*				
TrichhydFOR	(5′-GTTTGGGATGGCCTCAGAAT-3′)	95 °C for 10 min followed by 36 cycles at 96° for 5 s, 53° for 3 s and 68° for 15 s, and final extension at 72 °C for 10 s.	[22]	1000 bp
TrichhydREV	(5′-AGCCGAAGATGTTGTCGAAT-3′)			

**Table 3:** Genetic diversity and polymorphism of ITS1/5.8S/ITS2, *Fe-hydrogenase* and 18S rRNA sequences of *Trichomonas* sp.

Molecular Marker	bp	N	VS	GC%	H	hd (Mean ± SD)	π (Mean ± SD)	K
ITS1-5.8S-ITS2 intergenic region	321	96	100	34	18	0.588 ± 0.059	0.03375 ± 0.00705	9.75417
*Fe-hydrogenase*	851	118	107	53.9	27	0.893 ± 0.013	0.02159 ± 0.00305	17.40417
18S rRNA	1351	45	98	48.9	13	0.804 ± 0.040	0.00927 ± 0.00302	11.80

N = number of sequences analyzed; VS = number of site variables; GC% = G + C content; h = number of haplotypes; hd = haplotype diversity; SD = standard deviation; π = nucleotide diversity (per site); K = number of nucleotide differences.

## Data Availability

The datasets generated and analyzed during the current study are available on the NCBI Genbank Nucleotide platform (https://www.ncbi.nlm.nih.gov/genbank/ accessed on 12 January 2026) and can be accessed through the following accession numbers: PQ736456, PQ740175–PQ740209 for the ITS1-5.8S rRNA-ITS2 region; PQ762073–PQ762142 for the *iron hydrogenase* gene; PQ721469–PQ721475 for the 18S rRNA gene.

## Supplementary Materials

The following supporting information can be downloaded at https://www.mdpi.com/article/10.3390/pathogens15040428/s1. Video S1: Motile trophozoites of *Trichomonas gallinae* observed after 48 h of culture. Video S2: Motile trophozoites of *Trichomonas gallinae* observed after 72 h of culture; Supplementary Table S1. Detection results for *β-actin*, ITS-1-5.8S-ITS2, 18S rRNA, culture, and *Fe-hydrogenase* assays. Symbols indicate the following: (+) positive result; (−) negative result; (ND) not done; Supplementary Table S2. BLASTn results of sequences obtained in PCR assays for *Trichomonas* spp. from birds captured in Ribeirão Preto, Franca, Jundiaí, and Botucatu, Brazil.
