# Supplementary material for: Occurrence and Genetic Diversity of Trichomonas gallinae in Captive Synanthropic Birds in Southeastern Brazil
Source: Pathogens. 2026 Apr 16;15(4):428. doi: 10.3390/pathogens15040428 (PMC13119051; doi:10.3390/pathogens15040428)
Supplement: Supplementary file 1 [file pathogens-15-00428-s001.zip › Table S1 and Table S2 .pdf]

Supplementary Table S1. Detection results for B-ACTIN, ITS, 18S, culture, and *Fe-hidrogenase* assays. Symbols indicate: (+) positive result; (−) negative result; (ND) not done.

	Identification	Local	β-actin	ITS1/5.8S/ITS2	18S rRNA	Culture	*Fe-hidrogenase*
Ramphastos toco	J1	Jundíai	+	-	-	-	-
Ramphastos toco	J2	Jundíai	+	-	-	-	-
Ramphastos toco	J3	Jundíai	+	-	-	-	-
Ramphastos toco	J4	Jundíai	+	-	-	-	-
Ramphastos toco	J5	Jundíai	+	-	-	-	-
Ramphastos toco	J6	Jundíai	+	-	-	-	-
Ramphastos toco	J7	Jundíai	+	-	-	-	-
Ramphastos toco	J8	Jundíai	+	-	-	-	-
Ramphastos toco	J9	Jundíai	+	-	-	-	-
Nyctibius griseus	J10	Jundíai	+	-	-	-	-
Athene cunicularia	J11	Jundíai	+	-	-	-	-
Megascops choliba	J12	Jundíai	+	-	-	-	-
Megascops choliba	J13	Jundíai	+	-	-	-	-
Megascops choliba	J14	Jundíai	+	-	-	-	-
Columba livia	J15	Jundíai	+	-	-	-	-
Columba livia	J16	Jundíai	+	-	-	-	-
Columba livia	J17	Jundíai	+	-	-	-	-
Columba livia	J18	Jundíai	+	-	-	-	-
Rhynchotus rufescens	J19	Jundíai	+	-	-	-	-
Psittacara leucophthalmus	J20	Jundíai	+	-	-	-	-
Eupsittula aurea	J21	Jundíai	+	-	-	-	-
Psittacara leucophthalmus	J22	Jundíai	+	-	-	-	-
Psittacara leucophthalmus	J23	Jundíai	+	-	-	-	-
Psittacara leucophthalmus	J24	Jundíai	+	-	-	-	-
Amazona aestiva	J25	Jundíai	+	-	-	-	-
Ramphastos toco	J26	Jundíai	+	-	-	-	-
Ramphastos toco	J27	Jundíai	+	-	-	-	-
Rupornis magnirostris	J28	Jundíai	+	-	-	-	-
Rupornis magnirostris	J29	Jundíai	+	-	-	-	-
Ictinia plumbea	J30	Jundíai	-	-	-	-	-
Coragyps atratus	J31	Jundíai	+	-	-	-	-
Tigrisoma lineatum	J32	Jundíai	+	-	-	-	-
Columbina talpacoti	J33	Jundíai	+	-	-	-	-
Columbina talpacoti	J34	Jundíai	+	-	-	-	-
Columbina talpacoti	J35	Jundíai	+	-	-	-	-
Columbina talpacoti	J36	Jundíai	+	-	-	-	-
Columbina talpacoti	J37	Jundíai	+	-	-	-	-
Columbina talpacoti	J38	Jundíai	+	-	-	-	-
Columbina talpacoti	J39	Jundíai	+	-	-	-	-
Megascops choliba	J40	Jundíai	+	-	-	-	-
Athene cunicularia	J41	Jundíai	+	-	-	-	-
Megascops choliba	J42	Jundíai	+	-	-	-	-
Athene cunicularia	J43	Jundíai	+	-	-	-	-
Falco sparverius	J44	Jundíai	+	-	-	-	-

Falco sparverius	J45	Jundíai	+	-	-	-	-
Falco sparverius	J46	Jundíai	+	-	-	-	-
Falco sparverius	J47	Jundíai	+	-	-	-	-
Falco sparverius	J48	Jundíai	+	-	-	-	-
Falco sparverius	J49	Jundíai	+	-	-	-	-
Falco sparverius	J50	Jundíai	+	-	-	-	-
Coragyps atratus	J51	Jundíai	-	-	-	-	-
Coragyps atratus	J52	Jundíai	+	-	-	-	-
Coragyps atratus	J53	Jundíai	+	-	-	-	-
Pulsatrix perspicillata	J54	Jundíai	+	-	-	-	-
Asio clamator	J55	Jundíai	+	-	-	-	-
Caracara plancus	J56	Jundíai	+	-	-	-	-
Caracara plancus	J57	Jundíai	+	-	-	-	-
Ardea cocoi	J58	Jundíai	+	-	-	-	-
Sicalis flaveola	B01	Botucatu	+	-	-	-	-
Sporophila maximiliani	B02	Botucatu	+	-	-	-	-
Sporophila maximiliani	B03	Botucatu	+	-	-	-	-
Cyanoloxia brissonii	B04	Botucatu	+	-	-	-	-
Cyanoloxia brissonii	B05	Botucatu	+	-	-	-	-
Coryphospingus cucullatus	B06	Botucatu	+	-	-	-	-
Coryphospingus cucullatus	B07	Botucatu	+	-	-	-	-
Sporophila angolensis	B08	Botucatu	+	-	-	-	-
Saltator similis	B09	Botucatu	+	-	-	-	-
Gnorimopsar chopi	B10	Botucatu	+	-	-	-	-
Sporophila caerulescens	B11	Botucatu	+	-	-	-	-
Pitangus sulphuratus	B12	Botucatu	+	-	-	-	-
Pitangus sulphuratus	B13	Botucatu	+	-	-	-	-
Thraupis sayaca	B14	Botucatu	+	-	-	-	-
Chaetura meridionalis	B15	Botucatu	+	-	-	-	-
Chaetura meridionalis	B16	Botucatu	+	-	-	-	-
Sporophila caerulescens	B17	Botucatu	+	-	-	-	-
Sporophila caerulescens	B18	Botucatu	+	-	-	-	-
Columbina talpacoti	B19	Botucatu	+	-	-	-	-
Columbina talpacoti	B20	Botucatu	+	-	-	-	-
Ramphastos toco	B21	Botucatu	+	-	-	-	-
guira guira	B22	Botucatu	+	-	-	-	-
Brotogeris chiriri	B23	Botucatu	+	-	-	-	-
Anas bahamensis	B24	Botucatu	-	-	-	-	-
Athene cunicularia	B25	Botucatu	+	-	-	-	-
Tyto furcata	B26	Botucatu	+	-	-	-	-
Ramphastos toco	B27	Botucatu	+	-	-	-	-
Ramphastos toco	B28	Botucatu	+	-	-	-	-
Megascops choliba	B29	Botucatu	+	-	-	-	-
Megascops choliba	B30	Botucatu	+	-	-	-	-
Megascops choliba	B31	Botucatu	+	-	-	-	-
Megascops choliba	B32	Botucatu	+	-	-	-	-
Columba livia	RP01	Ribeirão Preto	+	-	-	-	-

Columba livia	RP02	Ribeirão Preto	+	-	-	-	-
Columba livia	RP03	Ribeirão Preto	+	-	-	-	-
Saltator similis	RP04	Ribeirão Preto	+	-	-	-	-
Saltator similis	RP05	Ribeirão Preto	+	-	-	-	-
Saltator similis	RP06	Ribeirão Preto	+	-	-	-	-
Cacicus cela	RP07	Ribeirão Preto	+	-	-	-	-
Turdus rufiventris	RP08	Ribeirão Preto	+	-	-	-	-
Aratinga jandaya	RP09	Ribeirão Preto	+	-	-	-	-
Thraupis sayaca	RP10	Ribeirão Preto	-	-	-	-	-
Eupsittula aurea)	RP11	Ribeirão Preto	+	-	-	-	-
Aratinga auricapillus	RP12	Ribeirão Preto	+	-	-	-	-
Aratinga auricapillus	RP13	Ribeirão Preto	+	-	-	-	-
Pyrrhura frontalis	RP14	Ribeirão Preto	+	-	-	-	-
Pyrrhura frontalis	RP15	Ribeirão Preto	+	-	-	-	-
Pyrrhura frontalis	RP16	Ribeirão Preto	+	-	-	-	-
Athene cunicularia	RP17	Ribeirão Preto	+	-	-	-	-
Ramphastos toco	RP18	Ribeirão Preto	+	-	-	-	-
Athene cunicularia	RP19	Ribeirão Preto	+	-	-	-	-
Tyto furcata	RP20	Ribeirão Preto	+	-	-	-	-
Falco sparverius	RP21	Ribeirão Preto	+	-	-	-	-
Saltator similis)	RP22	Ribeirão Preto	+	-	-	-	-
Columba livia	RP23	Ribeirão Preto	+	-	-	-	-
Columba livia	RP24	Ribeirão Preto	+	-	-	-	-
Rupornis magnirostris	RP25	Ribeirão Preto	+	-	-	-	-
Rupornis magnirostris	RP26	Ribeirão Preto	+	-	-	-	-
Rupornis magnirostris	RP27	Ribeirão Preto	+	-	-	-	-
Caracara plancus	RP28	Ribeirão Preto	+	-	-	-	-
Tyto furcata	RP29	Ribeirão Preto	+	-	-	-	-

Tyto furcata	RP30	Ribeirão Preto	+	-	-	-	-
Columba livia	RP31	Ribeirão Preto	+	-	-	-	-
Ara ararauna	RP32	Ribeirão Preto	+	-	-	-	-
Ramphastos toco	RP33	Ribeirão Preto	+	-	-	-	-
Coragyps atratus	RP34	Ribeirão Preto	+	-	-	-	-
Coragyps atratus	RP35	Ribeirão Preto	+	-	-	-	-
Coragyps atratus	RP36	Ribeirão Preto	+	-	-	-	-
Coragyps atratus	RP37	Ribeirão Preto	+	+	-	-	-
Coragyps atratus	RP38	Ribeirão Preto	+	-	-	-	-
Tyto furcata	RP39	Ribeirão Preto	+	-	-	-	-
Tyto furcata	RP40	Ribeirão Preto	+	-	-	-	-
Columba livia	RP41	Ribeirão Preto	+	-	-	-	-
Columba livia	RP42	Ribeirão Preto	+	-	-	-	-
Columba livia	RP43	Ribeirão Preto	+	-	-	-	-
Columba livia	RP44	Ribeirão Preto	+	-	-	-	-
Megascops choliba)	RP45	Ribeirão Preto	+	-	-	-	-
Nyctibius griseus	RP46	Ribeirão Preto	+	-	-	-	-
Athene cunicularia	RP47	Ribeirão Preto	+	-	-	-	-
Athene cunicularia	RP48	Ribeirão Preto	+	-	-	-	-
Pitangus sulphuratus	RP49	Ribeirão Preto	+	-	-	-	-
Turdus rufiventris	RP50	Ribeirão Preto	+	-	-	-	-
Pitangus sulphuratus	RP51	Ribeirão Preto	+	-	-	-	-
Columba livia domestica	F01	Franca	+	+	+	-	+
Columba livia domestica	F02	Franca	+	-	-	-	-
Columba livia domestica	F03	Franca	+	+	+	-	+
Columba livia domestica	F04	Franca	+	+	+	+	+
Columba livia domestica	F05	Franca	+	-	-	+	-
Columba livia domestica	F06	Franca	+	+	+	+	+
Columba livia domestica	F07	Franca	+	+	+	+	+
Columba livia domestica	F08	Franca	+	+	+	+	-
Columba livia domestica	F09	Franca	+	+	+	-	+
Columba livia domestica	F10	Franca	+	+	+	+	+
Columba livia domestica	F11	Franca	+	+	+	+	+

Columba livia domestica	F12	Franca	+	+	+	-	+
Columba livia domestica	F13	Franca	+	+	+	-	+
Columba livia domestica	F14	Franca	+	+	+	+	+
Columba livia domestica	F15	Franca	+	+	+	+	+
Columba livia domestica	F16	Franca	+	+	+	+	+
Columba livia domestica	F17	Franca	+	+	+	+	+
Columba livia domestica	F18	Franca	+	+	+	+	+
Columba livia domestica	F19	Franca	+	+	+	+	-
Columba livia domestica	F20	Franca	+	+	-	-	+
Columba livia domestica	F21	Franca	+	+	+	-	+
Columba livia domestica	F22	Franca	+	+	+	+	+
Columba livia domestica	F23	Franca	+	+	+	+	+
Columba livia domestica	F24	Franca	+	+	-	-	-
Columba livia domestica	F25	Franca	+	+	-	-	-
Columba livia domestica	F26	Franca	+	+	+	+	-
Columba livia domestica	F27	Franca	+	+	+	+	+
Columba livia domestica	F28	Franca	+	+	+	+	+
Columba livia domestica	F29	Franca	+	+	+	+	+
Columba livia domestica	F30	Franca	+	+	-	-	+
Columba livia domestica	F31	Franca	+	+	+	-	+
Columba livia domestica	F32	Franca	+	-	-	+	-
Columba livia domestica	F33	Franca	+	-	-	+	+
Columba livia domestica	F34	Franca	+	+	+	-	+
Columba livia domestica	F35	Franca	+	+	+	+	+
Columba livia domestica	F36	Franca	+	+	+	-	+
Columba livia domestica	F37	Franca	+	+	+	+	-
Columba livia domestica	F38	Franca	+	+	+	+	-
Columba livia domestica	F39	Franca	+	+	+	+	+
Columba livia domestica	F40	Franca	+	+	+	+	-
Columba livia domestica	F41	Franca	+	+	+	+	+
Columba livia domestica	F42	Franca	+	+	+	+	+
Columba livia domestica	F43	Franca	-	+	+	+	-
Columba livia domestica	F44	Franca	-	+	+	-	-
Columba livia domestica	F45	Franca	+	+	+	-	-
Columba livia domestica	F46	Franca	+	+	+	-	+
Columba livia domestica	F47	Franca	+	+	-	-	-
Columba livia domestica	F48	Franca	+	+	+	+	+
Columba livia domestica	F49	Franca	-	-	-	-	-
Columba livia domestica	F50	Franca	+	+	+	+	+
Columba livia domestica	F51	Franca	+	-	-	+	-
Columba livia domestica	F52	Franca	-	-	-	+	-
Columba livia domestica	F53	Franca	+	+	+	-	+
Columba livia domestica	F54	Franca	+	+	+	-	+
Columba livia domestica	F55	Franca	+	-	+	+	-
Columba livia domestica	F56	Franca	+	-	-	+	-
Columba livia domestica	F57	Franca	+	-	+	+	-
Columba livia domestica	F58	Franca	+	+	+	+	+
Columba livia domestica	F59	Franca	+	-	+	+	-

Columba livia domestica	F60	Franca	+	-	-	-	-
Columba livia domestica	F61	Franca	+	-	-	+	-
Columba livia domestica	F62	Franca	+	+	+	+	+
Columba livia domestica	F63	Franca	+	+	+	+	+
Columba livia domestica	F64	Franca	+	-	-	-	-
Columba livia domestica	F65	Franca	+	-	-	+	-
Columba livia domestica	F66	Franca	+	+	+	+	+
Columba livia domestica	F67	Franca	+	-	-	+	-
Columba livia domestica	F68	Franca	+	-	-	+	-
Columba livia domestica	F69	Franca	+	-	-	-	-
Columba livia domestica	F70	Franca	+	+	-	+	-
Columba livia domestica	F71	Franca	-	-	-	-	-
Columba livia domestica	F72	Franca	-	-	+	+	-
Columba livia domestica	F73	Franca	-	+	+	-	-
Columba livia domestica	F74	Franca	+	+	+	+	+
Columba livia domestica	F75	Franca	+	+	+	-	+
Columba livia domestica	F76	Franca	+	+	+	-	+
Columba livia domestica	F77	Franca	-	-	+	-	-
Columba livia domestica	F78	Franca	+	+	+	-	+
Columba livia domestica	F79	Franca	+	+	+	-	+
Columba livia domestica	F80	Franca	+	-	+	+	-
Columba livia domestica	F81	Franca	+	+	+	+	+
Columba livia domestica	F82	Franca	+	+	-	-	+
Columba livia domestica	F83	Franca	+	+	+	+	+
Columba livia domestica	F84	Franca	+	+	+	-	+
Columba livia domestica	F85	Franca	+	+	+	-	+
Columba livia domestica	F86	Franca	+	+	-	+	-
Columba livia domestica	F87	Franca	+	+	+	+	-
Columba livia domestica	F88	Franca	+	+	+	+	+
Columba livia domestica	F89	Franca	+	+	-	+	+
Columba livia domestica	F90	Franca	+	+	-	+	+
Columba livia domestica	F91	Franca	+	+	-	+	+
Columba livia domestica	F92	Franca	+	+	-	-	-
Columba livia domestica	F93	Franca	+	+	-	+	-
Columba livia domestica	F94	Franca	+	-	-	-	-
Columba livia domestica	F95	Franca	+	-	-	-	-
Columba livia domestica	F96	Franca	+	-	-	-	-
Columba livia domestica	F97	Franca	+	-	-	-	+
Columba livia domestica	F98	Franca	+	-	-	+	+
Columba livia domestica	F99	Franca	+	-	-	+	+
Columba livia domestica	F100	Franca	+	-	-	-	-
Columba livia domestica	F101	Franca	+	-	-	-	+
Columba livia domestica	F102	Franca	+	-	-	+	+
Columba livia domestica	F103	Franca	+	-	-	-	+
Columba livia domestica	F104	Franca	+	-	-	+	+
Columba livia domestica	F105	Franca	-	-	-	+	-
Columba livia domestica	F106	Franca	+	-	-	+	+
Columba livia domestica	F107	Franca	+	-	-	+	+

Columba livia domestica	F108	Franca	+	-	-	-	+
Columba livia domestica	F109	Franca	+	-	-	+	+
Columba livia domestica	F110	Franca	+	-	-	+	-
Columba livia domestica	F111	Franca	+	+	+	+	+
Columba livia domestica	F112	Franca	+	-	-	+	-
Columba livia domestica	F113	Franca	+	-	-	+	+
Columba livia domestica	F114	Franca	+	-	-	-	-
Columba livia domestica	F115	Franca	+	-	-	-	-
Columba livia domestica	F116	Franca	+	-	-	+	-
Columba livia domestica	F117	Franca	+	-	-	+	-
Columba livia domestica	F118	Franca	+	-	-	-	-
Columba livia domestica	F119	Franca	+	-	-	-	-
Columba livia domestica	F120	Franca	+	-	-	-	-
Columba livia domestica	F121	Franca	+	-	-	-	-
Columba livia domestica	JA962	SEPAS	+	+	-	ND	-
Ramphastos toco	JA02	SEPAS	-	-	-	ND	-
Columba livia domestica	JA03	SEPAS	+	+	-	ND	-
Tyto furcata	JA04	SEPAS	+	-		ND	-
Columba livia domestica	JA05	SEPAS	+	-	-	ND	-
Columba livia domestica	JA06	SEPAS	+	-	-	ND	-
Columba livia domestica	JA889	SEPAS	+	+	-	ND	-
Vanellus chilensis	JA08	SEPAS	+	-	-	ND	-
Forpus xanthopterygius	JA09	SEPAS	+	-	-	ND	-
Ramphastos toco	JA10	SEPAS	+	-	-	ND	-
Columba livia domestica	JA11	SEPAS	+	-	-	ND	-
Ramphastos toco	JA12	SEPAS	+	-	-	ND	-
Columba livia domestica	JA13	SEPAS	+	-	-	ND	-
Columba livia domestica	JA14	SEPAS	+	-	-	ND	-
Columba livia domestica	JA15	SEPAS	+	-	-	ND	-
Columba livia domestica	JA16	SEPAS	+	-	-	ND	-
Columba livia domestica	JA17	SEPAS	+	-	-	ND	-
Columba livia domestica	JA18	SEPAS	+	-	-	ND	-
Ara ararauna	JA19	SEPAS	+	-	-	ND	-

Supplementary Table S2. BLASTn results of sequences obtained in PCR assays for *Trichomonas* spp. from birds captured in Ribeirão Preto, Franca, Jundiaí, and Botucatu, Brazil.

N°	Species	Gene	Sequence size (bp)	*Query cover* (%)	*E-value*	Identity (%)	GenBank sequence
1	*Columba livia domestica* Franca	Region ITS1/5.8 S/ITS2	368	100%	0	100%	*Trichomonas sp. detected in a Columba livia in China*. (MH733819)
2	*Columba livia domestica* Franca	Region ITS1/5.8 S/ITS2	376	100%	0	100%	*Trichomonas sp. detected in a Columba livia in China*. (MH733819)
3	*Columba livia domestica* Franca	Region ITS1/5.8 S/ITS2	376	100%	0	100%	*Trichomonas sp. detected in a Columba livia in China*. (H733819)
4	*Columba livia domestica* Franca	Region ITS1/5.8 S/ITS2	372	100%	0	100%	*Trichomonas sp. detected in a Columba livia in China*. (MH733819)
5	*Columba livia domestica* Franca	Region ITS1/5.8 S/ITS2	387	100%	0	100%	*Trichomonas sp. detected in a Columba livia in China*. (MH733819)
6	*Columba livia domestica* Franca	Region ITS1/5.8 S/ITS2 Region ITS1/5.8 S/ITS2	383	100%	0	100%	*Tetratrichomonas gallinarum* (MK770861)
7	*Columba livia domestica* Franca	Region ITS1/5.8 S/ITS2	367	100%	0	100%	*Trichomonas sp. detected in a Columba livia in China*. (MH733819)
8	*Columba livia domestica* Franca	Region ITS1/5.8 S/ITS2	386	100%	0	100%	*Trichomonas sp. detected in a Columba livia in China*. (MH733819)

9	*Columba livia domestica* Franca	Region ITS1/5.8 S/ITS2	386	100%	0	100%	*Trichomonas sp. detected in a Columba livia in China*. (MH733819)
10	*Columba livia domestica* Franca	Region ITS1/5.8 S/ITS2	377	100%	0	100%	*Trichomonas sp. detected in a Columba livia in China*. (MH733819)
11	*Columba livia domestica* Franca	Region ITS1/5.8 S/ITS2	370	100%	0	100%	*Trichomonas sp. detected in a Columba livia in* Spain. (EU881912)
12	*Columba livia domestica* Franca	Region ITS1/5.8 S/ITS2	383	100%	0	100%	*Tetratrichomonas gallinarum* (MK770861)
13	*Columba livia domestica* Franca	Region ITS1/5.8 S/ITS2	376	100%	0	100%	*Trichomonas sp. detected in a Columba livia in China* (MH733819)
14	*Columba livia domestica* Franca	Region ITS1/5.8 S/ITS2	378	100%	0	100%	*Trichomonas sp. detected in a Columba livia in China* (MH733819)
15	*Columba livia domestica* Franca	Region ITS1/5.8 S/ITS2	384	100%	0	100%	*Tetratrichomonas gallinarum* (MK770861)
16	*Columba livia domestica* Franca	Region ITS1/5.8 S/ITS2	370	100%	0	100%	*Tetratrichomonas gallinarum* (MK770861)
17	*Columba livia domestica* Franca	Region ITS1/5.8 S/ITS2	340	100%	0	100%	*Tetratrichomonas gallinarum* (MK770861)
18	*Columba livia domestica* Franca	Region ITS1/5.8 S/ITS2	400	100%	0	100%	*Tetratrichomonas gallinarum* (MK770861)
19	*Columba livia domestica* Franca	Region ITS1/5.8 S/ITS2	371	100%	0	100%	*Trichomonas sp. detected in a Columba livia in China* (MH733819)
20	*Columba livia domestica* Franca	Region ITS1/5.8 S/ITS2	299	100%	3e-142	99,65%	*Trichomonas sp. detected in a Streptopelia turtur* in the United Kingdom. (MT632478)

21	*Columba livia domestica* Franca	Region ITS1/5.8 S/ITS2	376	100%	0	100%	*Trichomonas sp. detected in a Columba livia in China* (MH733819)
22	*Columba livia domestica* Franca	Region ITS1/5.8 S/ITS2	377	100%	0	100%	*Trichomonas sp. detected in a Columba livia in China* (MH733819)
23	*Columba livia domestica* Franca	Region ITS1/5.8 S/ITS2	384	100%	0	100%	*Tetratrichomonas gallinarum* (MK770861)
24	*Columba livia domestica* Franca	Region ITS1/5.8 S/ITS2	385	100%	0	100%	*Trichomonas sp. detected in a Columba livia in China* (MH733819)
25	*Columba livia domestica* Franca	Region ITS1/5.8 S/ITS2	410	100%	0	99,73%	*Trichomonas sp. detected in a Columba livia in* Spain (EU881912)
26	*Columba livia domestica* Franca	Region ITS1/5.8 S/ITS2	409	100%	0	100%	*Trichomonas sp. detected in a Columba livia in China* (MH733819)
27	*Columba livia domestica* Franca	Region ITS1/5.8 S/ITS2	255	100%	2e-128	100%	*Trichomonas sp. detected in a Columba livia in China* (MH733821.1)
28	*Columba livia domestica* Franca	Region ITS1/5.8 S/ITS2	366	100%	0	100%	*Trichomonas sp. detected in a Columba livia in China* (MH733819.1)
29	*Columba livia domestica* Franca	Region ITS1/5.8 S/ITS2	366	100%	0	100%	*Trichomonas sp. detected in a Columba livia in China* (MH733819.1)
30	*Columba livia domestica* Franca	Region ITS1/5.8 S/ITS2	358	100%	0	100%	*Trichomonas sp. detected in a Columba livia in China* (MH733819.1)
31	*Columba livia domestica* Franca	Region ITS1/5.8 S/ITS2	359	100%	0	100%	*Tetratrichomonas gallinarum* (MK770861)

32	*Columba livia domestica* Franca	Region ITS1/5.8 S/ITS2	351	100%	0	100%	*Tetratrichomonas gallinarum* (MK770861)
33	*Columba livia domestica* Jaboticabal	Region ITS1/5.8 S/ITS2	339	99%	1e-172	99,71%	*Trichomonas sp. detected in a Ramphastos dicolorus*. (ON000429.1)
34	*Columba livia domestica* Cultivation	Region ITS1/5.8 S/ITS2	309	95%	4e-151	100%	*Trichomonas sp. detected in a Columba livia in China* (MH733821.1)
35	*Columba livia domestica* Franca	Region ITS1/5.8 S/ITS2	358	100%	0	100%	*Trichomonas sp. detected in a Columba livia in China* (MH733819.1)
36	*Columba livia domestica* Franca	*Fe-hidrogen ase*	850	100%	0	100%	*Trichomonas gallinae* (KP900038)- Detected on Streptopelia decaocto in Spain
37	*Columba livia domestica* Franca	*Fe-hidrogen ase*	817	100%	0	95,51%	*Trichomonas gallinae (KP900038)*- Detected on Streptopelia decaocto in Spain
38	*Columba livia domestica* Franca	*Fe-hidrogen ase*	777	100%	0	99,49%	*Trichomonas gallinae* (KX514374)- Detected on Columba palumbus in Spain
38	*Columba livia domestica* Franca	*Fe-hidrogen ase*	811	100%	0	99,38%	*Trichomonas gallinae* (KX514374)- Detected on Columba palumbus in Spain
39	*Columba livia domestica* Franca	*Fe-hidrogen ase*	822	100%	0	100%	*Trichomonas gallinae* (KP900038)- detectado em *Streptopelia decaocto* na Espanha

40	*Columba livia domestica* Franca	*Fe-hidrogenase*	809	100%	0	100%	*Trichomonas gallinae* (KX514374)-Detected on *Columba palumbus* in Spain
41	*Columba livia domestica* Franca	*Fe-hidrogenase*	867	100%	0	99,77%	*Trichomonas gallinae* (KP900040)-Detected on *Columba livia* in Spain
42	*Columba livia domestica* Franca	*Fe-hidrogenase*	777	100%	0	100%	*Trichomonas gallinae* (KX514374)-Detected on *Columba palumbus* in Spain
43	*Columba livia domestica* Franca	*Fe-hidrogenase*	817	100%	0	99,76%	*Trichomonas gallinae* (KP900040)-Detected on *Columba palumbus* in Spain
44	*Columba livia domestica* Franca	*Fe-hidrogenase*	820	100%	0	100%	*Trichomonas gallinae* (AF446077)
45	*Columba livia domestica* Franca	*Fe-hidrogenase*	811	100%	0	100%	*Trichomonas gallinae* (KX514374) Detected on *Columba palumbus* in Spain
46	*Columba livia domestica* Franca	*Fe-hidrogenase*	859	100%	0	99.65%	*Trichomonas gallinae* (KP900040) Detected on *Columba livia* in Spain
47	*Columba livia domestica* Franca	*Fe-hidrogenase*	823	100%	0	100%	*Trichomonas gallinae (KP900038)-Detected on Streptopelia decaocto in Spain*

48	*Columba livia domestica* Franca	*Fe-hidrogenase*	830	100%	0	100%	*Trichomonas gallinae* (KP900038)- Detected on Streptopelia decaocto in Spain
49	*Columba livia domestica* Franca	*Fe-hidrogenase*	895	100%	0.0	99.11%	*Trichomonas gallinae* (KP900038)- Detected on Streptopelia decaocto in Spain
50	*Columba livia domestica* Franca	*Fe-hidrogenase*	837	100%	0.0	99.76%	*Trichomonas gallinae* (KP900040)- d Detected on *Columba livia* in Spain
51	*Columba livia domestica* Franca	*Fe-hidrogenase*	826	100%	0.0	99.76%	*Trichomonas gallinae* (KP900040)- Detected on *Columba livia* in Spain
52	*Columba livia domestica* Franca	*Fe-hidrogenase*	838	100%	0.0	99.40%	*Trichomonas gallinae* (KP900038)- Detected on *Streptopelia decaocto* in Spain
53	*Columba livia domestica* Franca	*Fe-hidrogenase*	810	100%	0	100%	*Trichomonas gallinae* (AF446077)
54	*Columba livia domestica* Franca	*Fe-hidrogenase*	862	100%	0.0	99.65%	*Trichomonas gallinae* (KP900040)- Detected on *Columba livia* in Spain
55	*Columba livia domestica* Franca	*Fe-hidrogenase*	814	100%	0.0	99.51%	*Trichomonas gallinae* (KP900038)- Detected on *Streptopelia decaocto* in Spain
56	*Columba livia domestica* Franca	*Fe-hidrogenase*	598	100%	0	100%	*Trichomonas gallinae* (KX514374) Detected on *Columba palumbus* in

							Spain
57	*Columba livia domestica* Franca	*Fe-hidrogenase*	842	100%	0.0	99.29%	*Trichomonas gallinae* (KP900038)- Detected on *Streptopelia decaocto* in Spain
58	*Columba livia domestica* Franca	*Fe-hidrogenase*	876	99%	0.0	99.43%	*Trichomonas gallinae* (KP900034)- Detected on *Streptopelia decaocto* in Spain
59	*Columba livia domestica* Franca	*Fe-hidrogenase*	836	100%	0.0	99.76%	*Trichomonas gallinae* (KP900040)- Detected on *Columba livia* in Spain
60	*Columba livia domestica* Franca	*Fe-hidrogenase*	862	100%	0	100%	*Trichomonas gallinae* (AF446077)
61	*Columba livia domestica* Franca	*Fe-hidrogenase*	905	98%	0	100%	*Trichomonas gallinae* (KP900038)- Detected on *Streptopelia decaocto* in Spain
62	*Columba livia domestica* Franca	*Fe-hidrogenase*	843	100%	0	100%	*Trichomonas gallinae* (AF446077)
63	*Columba livia domestica* Franca	*Fe-hidrogenase*	872	100%	0	100%	*Trichomonas gallinae* (KP900038)- Detected on *Streptopelia decaocto* in Spain
64	*Columba livia domestica* Franca	*Fe-hidrogenase*	901	98%	0	100%	*Trichomonas gallinae* (AF446077)
65	*Columba livia domestica* Franca	*Fe-hidrogenase*	881	100%	0	100%	*Trichomonas gallinae* (AF446077)
66	*Columba livia domestica* Franca	*Fe-hidrogenase*	870	100%	0	99,66%	*Trichomonas gallinae* (KP900040)- Detected on *Columba livia* in Spain

67	*Columba livia domestica* Franca	*Fe-hidrogenase*	890	100%	0	99,66%	*Trichomonas gallinae* (KP900040)- Detected on *Columba livia* in Spain
68	*Columba livia domestica* Franca	*Fe-hidrogenase*	819	100%	0	99,39%	*Trichomonas gallinae* (KP900038)- Detected on *Streptopelia decaocto* in Spain
69	*Columba livia domestica* Franca	*Fe-hidrogenase*	741	100%	0	99,46%	*Trichomonas gallinae* (KX514374) Detected on *Columba palumbus* in Spain
70	*Columba livia domestica* Franca	*Fe-hidrogenase*	855	100%	0	99,77%	*Trichomonas gallinae (KP900040)*- Detected on *Columba livia in* Spain
71	*Columba livia domestica* Franca	*Fe-hidrogenase*	862	100%	0	100%	*Trichomonas gallinae (KP900038)*- Detected on *Streptopelia decaocto* in Spain
72	*Columba livia domestica* Franca	*Fe-hidrogenase*	850	100%	0	100%	*Trichomonas gallinae* (KP900038)- Detected on *Streptopelia decaocto* in Spain
73	*Columba livia domestica* Franca	*Fe-hidrogenase*	829	100%	0	100%	*Trichomonas gallinae* (AF446077)
74	*Columba livia domestica* Franca	*Fe-hidrogenase*	865	100%	0	99,70%	*Trichomonas gallinae* (KP900040)- Detected on *Columba livia in* Spain
75	*Columba livia domestica* Franca	*Fe-hidrogenase*	849	100%	0	100%	*Trichomonas gallinae* (AF446077)

76	*Columba livia domestica* Franca	*Fe-hidrogenase*	859	98%	0	99,41%	*Trichomonas gallinae* (KP900034)-Detected on *Streptopelia decaocto* in Spain
77	*Columba livia domestica* Franca	*Fe-hidrogenase*	743	100%	0	99,46%	*Trichomonas gallinae* (KX514374) Detected on *Columba palumbus* in Spain
78	*Columba livia domestica* Franca	*Fe-hidrogenase*	881	98%	0	99,43%	*Trichomonas gallinae* (KP900034)-Detected on *Streptopelia decaocto* in Spain
79	*Columba livia domestica* Franca	*Fe-hidrogenase*	855	100%	0	99,65%	*Trichomonas gallinae* (KP900040)-Detected on *Columba livia* in Spain
80	*Columba livia domestica* Franca	*Fe-hidrogenase*	862	100%	0	100%	*Trichomonas gallinae* (KP900038)-Detected on *Streptopelia decaocto* in Spain
81	*Columba livia domestica* Franca	*Fe-hidrogenase*	853	100%	0	99,77%	*Trichomonas gallinae* (KP900040)-Detected on *Columba livia* in Spain
82	*Columba livia domestica* Franca	*Fe-hidrogenase*	826	100%	0	99,52%	*Trichomonas gallinae* (KP900038)-Detected on *Streptopelia decaocto* in Spain
83	*Columba livia domestica* Franca	*Fe-hidrogenase*	863	100%	0	99,42%	*Trichomonas gallinae* (KP900038)-Detected on *Streptopelia decaocto* in Spain

84	*Columba livia domestica* Franca	*Fe-hidrogenase*	828	100%	0	99,76%	*Trichomonas gallinae* (KP900040)-Detected on *Columba livia* in Spain
85	*Columba livia domestica* Franca	*Fe-hidrogenase*	883	100%	0	99,66%	*Trichomonas gallinae* (KP900040)-Detected on *Columba livia* in Spain
86	*Columba livia domestica* Franca	*Fe-hidrogenase*	822	100%	0	100%	*Trichomonas gallinae* (KP900038)-Detected on *Streptopelia decaocto* in Spain
87	*Columba livia domestica* Franca	*Fe-hidrogenase*	880	100%	0	99,66%	*Trichomonas gallinae* (KP900040)-Detected on *Columba livia* in Spain
88	*Columba livia domestica* Franca	*Fe-hidrogenase*	821	100%	0	99,76%	*Trichomonas gallinae* (AF446077)
89	*Columba livia domestica* Franca	*Fe-hidrogenase*	832	100%	0	99,52%	*Trichomonas gallinae* (KP900038)-Detected on *Streptopelia decaocto* in Spain
90	*Columba livia domestica* Franca	*Fe-hidrogenase*	903	95%	0	100%	*Trichomonas gallinae* (AF446077)
91	*Columba livia domestica* Franca	*Fe-hidrogenase*	820	100%	0	100%	*Trichomonas gallinae* (KP900038)-Detected on *Streptopelia decaocto* in Spain
92	*Columba livia domestica* Franca	*Fe-hidrogenase*	850	99%	0	99,53%	*Trichomonas gallinae* (KP900038)-Detected on *Streptopelia decaocto* in Spain
93	*Columba livia domestica* Franca	*Fe-hidrogenase*	828	100%	0	100%	*Trichomonas gallinae* (AF446077)

94	*Columba livia domestica* Franca	*Fe-hidrogenase*	810	100%	0	100%	*Trichomonas gallinae* (AF446077)
95	*Columba livia domestica* Franca	*Fe-hidrogenase*	859	100%	0	100%	*Trichomonas gallinae (AF446077)*
96	*Columba livia domestica* Franca	18S rRNA	999	99%	0	100%	*Trichomonas gallinae* (MK932772)-Detected on a *Columba livia* in Portugal
97	*Columba livia domestica* Franca	18S rRNA	987	100%	0	100%	*Trichomonas gallinae* (MK932772)-Detected on a *Columba livia* in Portugal
98	*Columba livia domestica* Franca	18S rRNA	988	100%	0	99,90%	*Trichomonas gallinae* (MK932772)-Detected on a *Columba livia* in Portugal
99	*Columba livia domestica* Franca	18S rRNA	1000	99%	0	100%	*Trichomonas gallinae* (MK932772)-Detected on a *Columba livia* in Portugal
100	*Columba livia domestica* Franca	18S rRNA	888	100%	0	99,89%	*Trichomonas gallinae* (MK932772)-Detected on a *Columba livia* in Portugal
101	*Columba livia domestica* Franca	18S rRNA	1404	100%	0	99,86%	*Trichomonas gallinae* (EU215374)-Detected in *Eurasian dove* in Texas
102	*Columba livia domestica* Franca	18S rRNA	1331	100%	0	100%	*Trichomonas gallinae* (MK932772)-Detected on a *Columba livia* in Portugal
103	*Columba livia domestica* Franca	18S rRNA	1334	100%	0	100%	*Trichomonas gallinae* (MK932772)-

							Detected on a *Columba livia* in Portugal
10 4	*Columba livia domestica* Franca	18S rRNA	1372	100%	0	99,93%	*Trichomonas gallinae* (MK932772)- Detected on a *Columba livia* in Portugal
